# Book Reviews

**Published:** 1829-04

**Authors:** 


### CRITICAL ANALYSES.

Quæ laudanda forent, et quæ culpanda, vicissim

Illa, prius, cretâ; mox hæc, carbone, *notamus*.—Persius.

*Illustrations of the Diseases of the Breast.* By Sir Astley Cooper, Bart. F.R.S. Serjeant Surgeon to His Majesty; Consulting Surgeon of Guy's Hospital; Lecturer on Anatomy and Surgery, &c. In two Parts. Part I.—4to. pp. 89; with nine beautifully coloured Plates, including numerous Figures.—Longman and Co. Feb. 1829.

If we were to scrutinize the motives which lead to the publication of the great majority of books, we should probably conclude that the diligence of men is generally to be estimated by their eagerness for immediate reward, or their ambition of future fame. The continued industry of Sir Astley Cooper cannot be prompted by either of these motives. Of present reward he is independent; and, of future fame, so perfectly secure, that he might safely repose upon the high reputation he has established. His zeal must now be attributed to the honourable and philanthropic desire of benefiting the public, by communicating to his surgical brethren the results of his long and extensive experience.

The importance of the subject to which our attention is directed in this work, will not be denied. These Illustrations of the Diseases of the Breast are divided into two parts: those which are, and those which are not malignant. In this part the author has confined himself to the description of the latter, distinguishing those which do not arise from a vitiated state of the system, nor produce any dangerous constitutional effects, and do not contaminate the parts in their neighbourhood, nor affect those at a distance from their original seat. It is observed, however, that some of these swellings, when they have existed long in a dormant state, will have alterations produced in them by changes of the constitution, by which their extirpation may be rendered necessary; for malignancy may be lighted up in them by constitutional disease, by anxiety of mind, and by the cessation of the menstrual secretion. The female breast is liable to almost all the complaints of other structures, and to some which are peculiarly its own.

"The uninformed surgeon is too apt to fall in with the opinion of the vulgar, and to confound all the swellings of the breast under the general term of Cancer; and yet every surgeon who has fully investigated the character of these swellings, by examination of the diseased parts after operation, must be aware of the great variety which prevails in their nature and appearances, and is therefore led to the conclusion that, far from their being all of one family, a great number of genera of tumors actually exist. He will soon learn that some are the effect of acute inflammation; that others are of a simple chronic kind; that some are chronic accompanied with specific action; and that others are specific and malignant. It is, therefore, the surgeon's duty to discriminate these differences in the living body; and this he can only accomplish by a very careful and nice manipular examination of the complaint, by having repeatedly inspected the parts which have been removed in operations, by examining those which have been met with in the body after death, and by an accurate and minute history of the case. The experience arising from these different sources gives him the power of accurately judging of the nature of the disease when it is presented to his attention in the living body." (P. 2.)

Sir Astley divides the diseases of the breast into three classes: first, those which are the result of common inflammation, whether it be acute or chronic; secondly, into complaints which arise from peculiar or specific action, but which are not malignant, and do not contaminate other structures; thirdly, into those which are not only founded on local, malignant, and specific actions, but which are connected with a peculiar and unhealthy state of the constitution. By a malignant complaint is implied a local diseased action, which not only affects the parts in which it is originally situated, but which contaminates those in its neighbourhood. It is produced by a morbid state of the constitution, and is frequently accompanied by similar disease in other, and even remote, parts of the body.

The following extract describes the purport of the work, and at the same time may convey a useful lesson to the presumptuous many who conceive their decision to be infallible.

"It will be my object in the following pages, with the aid of engravings, to detail the symptoms, describe the external characters, and exhibit the internal appearances of each of these diseases, so far as I have been able to observe and examine them; and, in doing this, I shall endeavour to point out their discriminating marks, so as to enable the surgeon to distinguish them in the living body. I am fully aware of the difficulty of the task, and am ready to acknowledge that I have been often mistaken in my diagnosis; but if such errors of judgment occur to one who has had a considerable share of practice and experience, and trusts he has not been an idle or inattentive spectator of what has been presented to his observation, how often must those be liable to error who do not industriously investigate the nature of disease by dissection, and compare it with its external characters in the living body?" (P. 5.)

Chapter ii. "*On the Effects of common Inflammation in the Breast.*"—Acute inflammation in the breast differs little from the same inflammation in other parts of the body, excepting in the severity of suffering which it produces. It is adhesive in the first stage, suppurative in the second, and ulcerative in the third. It occurs most frequently in women at an early period after delivery, in consequence of the abundant determination of blood to the breasts for the secretion of milk. In the first stage, cold evaporating lotions and purgatives are to be employed: "but if the patient suffer from the cold produced by the evaporation of the spirit, a simple tepid poultice may be substituted for it, occasionally applying leeches to the swelling; still recollecting that the chief dependence is upon purging." If suppuration is not prevented by these means, warm poppy fomentations and poultices are to be employed. The late Dr. John Clarke objected to the use of warm applications in such cases; and, as his opinions have had much influence upon the practice generally adopted, we shall state his views, together with the result of our own observation. He contended that poultices and fomentations "derived a large quantity of blood to the parts, and that, by their relaxant power, they weakened the tone and strength of the parts to such a degree, that if matter should inevitably be formed, (which, when it happens, is generally in a large quantity,) the abscess is always very difficult of healing, especially if a large opening should be artificially made into it."* Instead, therefore, of such applications, Dr. Clarke advised the use of cold saturnine lotions; and he recommends us to continue them without intermission, even "if the breast should suppurate, and that the fluctuation of matter can be distinctly felt under the skin, until the abscess points."† We have tried, in many cases of milk abscess, the treatment advised by Sir Astley Cooper, and the contrary plan which is supported by the authority of Dr. Clarke. In whatever manner these cases may be treated, they will frequently prove tedious and painful: but, judging from our own experience, the objections urged by Dr. Clarke against the use of warm emollient applications

* Practical Essays on the Management of Pregnancy and Labour. By John Clarke, M.D. Page 42.

† Ibid. page 43.

are more imaginary than real. The continued use of cold lotions is generally very distressing to the patient; while, as Sir Astley observes, poppy fomentations and poultices sooth and relax the part, and, by their narcotic qualities, diminish the sensibility of the nerves

If much pain exists, opium and saline draughts will also be necessary. It is frequently a question with surgeons whether these abscesses should be opened, or be left to break spontaneously. Upon this subject Sir A. thus states his opinion:

"If the abscess be quick in its progress, if it be placed on the anterior surface of the breast, and if the sufferings which it occasions are not excessively severe, it is best to leave them to their natural course, rather than employ the lancet for the discharge of the matter. But if, on the contrary, the abscess in its commencement be very deeply placed, if its progress be tedious, if the local sufferings be excessively severe, if there be a high degree of irritative fever, and the patient suffer from profuse perspiration and want of rest, much time is saved, and a great diminution of suffering produced, by discharging the matter by the lancet. Still it is wrong to penetrate with the lancet through a thick covering of the abscess, as the opening does not succeed in establishing a free discharge of matter; for the aperture closes by adhesion, the accumulation of matter proceeds, and ulceration will still continue; on this account the opening should be made where the matter is most superficial and the fluctuation is distinct, and it should be in size proportioned to its depth." (P. 10.)

Sometimes several abscesses form in the same breast, quickly succeeding each other, and lead to very protracted suffering. Opium and quinine will here be required. Sinuses are sometimes formed. The best mode of treating these cases Sir Astley has found to be by injecting them with a solution of two or three drops of the strong sulphuric acid to an ounce of rosewater, and to cover the breast with the same solution.

"Now and then a deep-seated abscess forms between the posterior surface of the breast and the ribs, which, when it breaks, leaves a sinus which leads to the ribs. An exfoliation of part of the rib afterwards occurs, occasioning a very protracted suffering; and in these cases, as well as in the former, injecting the diluted acids is the best practice." (P. 11.)

The division of the sinus by the knife is unnecessary. In the former case it will heal by adhesion. In the latter, unless the exfoliating bone be loose, no advantage will be derived from the incision. Sir Astley once saw a lady of delicate constitution, who suffered much from mental anxiety: after her lying-in she had milk abscess, which broke and discharged freely; and then, instead of healing, the whole breast became excessively swollen, and a truly fungoid excrescence appeared. The patient was soon destroyed by this disease.

Hardness of the breast sometimes remains after abscesses for a considerable time.

"As a morbid action will sometimes, and at a very distant period, arise in the swelling, it is a great object to dissipate it quickly; which will be best effected by the application of the Emplastrum Ammoniaci cum Hydrargyro, or by rubbing the part with the Iodine ointment." As a general rule, it is best to continue the child at the breast as long as the mother can bear it.

Soreness of the nipples sometimes prevents women from suckling, and hence distention, inflammation, and abscess of the breast. To prevent these consequences, the breast should be drawn; but the sooner the child can be put to it again, the better. "The best application to the sore nipple is a solution of borax in water, in the proportion of a drachm of borax, three ounces and a half of water, and half an ounce of spirit of wine." Sir Astley also recommends, as a preventive of sore nipples, that women who have been subject to them should wash them, some time before the lying-in, with strong brine.

"*Of chronic Abscesses.*"—From chronic inflammation an abscess is sometimes produced, in which, from the length of time it is forming, from the little pain and the absence of redness and heat in the part, and from the want of rigors and other constitutional symptoms, the formation of matter is not suspected, and the swelling is supposed to be malignant, and to require an operation. In such cases Sir Astley has "seen the operation for removing the swelling begun, and in its progress, the knife having accidentally entered the abscess, the surgeon, by the escape of the matter, having been informed of his error, the operation was suspended; and a poultice being applied, the case ended favorably." For these chronic swellings mercurial alteratives are required, and the Empl. Ammon. cum Hydrarg. should be applied to the part.

A swelling is sometimes formed in the breast after a lying-in, which the author terms "the lacteal or lactiferous," because it arises from a large collection of milk in one of the lactiferous tubes.

"Its cause is a chronic inflammation of one of the lactiferous tubes near the nipple, by which its aperture becomes closed, and the tube obliterated to the extent of an inch or more. The patient applies to the surgeon some time after delivery with a swelling in the breast; unpreceded by the symptoms of abscess, it distinctly fluctuates, and she complains exceedingly of a sense of distention in the part; and, when the child is put to the breast to relieve it, the pain and distention are increased by the draught of milk which enters the breast so soon as the child begins to suck. The swelling is confined to one portion of the breast, from the nipple to the circumference of the organ, and it gives a distinct sense of fluctuation. The cutaneous veins are very large, but the part is otherwise undiscoloured. If a lancet be passed into the swelling, several ounces of milk are discharged; and the milk, being suffered to rest for a few hours, forms a cream upon its surface. If a slight puncture only be made, the milk be discharged, and the opening suffered to immediately close, the accumulation recommences, and in a short time the same appearances and sufferings are renewed.

"When the distention of the swelling is excessive, it sometimes ulcerates, and discharges the milk which it has contained, by a small aperture at a little distance from the nipple; and the opening so produced often continues through the whole period of suckling, the milk being lost, from the aperture not being received into the child's mouth: and this opening is difficult to heal, until, by weaning the child and by purges, the secretion of milk be entirely checked.

"The treatment which this case requires is as follows: If the mother be prevailed upon to wean her child, as the secretion of milk will soon cease in this obstructed part as in other parts of the breast, a simple puncture will suffice to relieve the distended tube of the milk which it contains. But, if the child still continue at the breast, the opening may be made larger, and the milk be suffered to escape at the artificial aperture whilst the child is sucking: thus imitating the natural relief which the ulcerative process sometimes produces, until the secretion of milk ceases, from the weaning of the child, and from purges to the mother." (P. 17.)

This disease resembles in its nature the ranula, excepting in the fluid secreted. The one is an obstruction of the submaxillary duct; the other is an obstructed lactiferous tube.

Chap. iii. "*On the Hydatid Disease of the Breast.*"—There are four species of these swellings, three of which are not malignant: one is of a malignant nature. The first species exists in the form of simple bags, which contain a serous fluid. Sir Astley would call them cellulous hydatids.

"The breast gradually swells, and in the beginning is entirely free from pain or tenderness; it becomes hard, and no fluctuation can then be discovered in it; it continues slowly growing for months, and even for years, sometimes acquiring very considerable magnitude, the largest I have seen having weighed nine pounds; but in other cases, although the bosom was quite filled with these bags, yet it never exceeded twice the size of the other breast.

"At first the swelling feels entirely solid, so that it bears a great resemblance to a simple chronic enlargement of the breast; but, after a great length of time, a fluctuation can at one part be discovered in it, and then the breast begins to increase more quickly; and in several parts similar fluctuations can be detected. The subcutaneous veins become varicose; but, although the breast is immensely enlarged, it still continues almost entirely free from pain: but to this there are exceptions, for some persons feel an unusual heat, and some, as the breast increases, suffer pain in the part and in the shoulder. The tumor is extremely moveable upon the pectoral muscle, is very pendulous; and in some cases the whole of the mammary gland, in others only a small portion of it, becomes involved in the disease. At length one of the fluctuating portions of the breast slowly inflames, ulcerates, and discharges a large quantity of serum, or of a fluid having its general character, but of a consistence somewhat more glairy; and the sac being emptied, and the external opening closed, if the fluid be entirely discharged, it is a long time before it reaccumulates; and sometimes the sides of the sac adhere, and the cyst ceases to secrete. In other instances I have known the swelling break, and discharge a mucilaginous fluid mixed with serum; and several of the cells in succession, and at distant periods, pass through the ulcerative process, and form sinuses, which are very difficult to heal.

"Excepting during the process of ulceration, the general health remains entirely undisturbed, and the person suffers so little, either locally or constitutionally, that her friends do not discover her malady; and nothing would lead her to consent to an operation for its removal, but the anxiety of mind and the apprehension which the idea of a cancer produces, and the great inconvenience and distress which the weight of a large swelling occasions.

"Although the whole breast should be involved in the disease, and even although the swelling ulcerates, discharges largely, and puts on a formidable appearance, and even becomes of the enormous size which will be seen in Plate the 2d, yet the glands in the axilla remain entirely free from disease; or, if one be slightly enlarged, it is from simple irritation only, and it disappears when the complaint in the breast is removed." (P. 20.)

Although, in the greater number of cases, the whole breast becomes involved in the disease, yet Sir A. has seen it several times affect one part only; and the removal of portions of the breast has been sufficient to prevent a return of the complaint.

*Diagnosis.*—"This disease, in its first stage, resembles simple chronic inflammation; but it may be distinguished from it by the absence of tenderness upon pressure, and the perfect health in which the patient remains stamps it to be an entirely local disease." In the second stage, the best criterion is the puncture of the bag, when the evacuation of a clear serum, instead of a purulent fluid, shows the nature of the disease.

*Treatment.*—No local applications are beneficial. The constitution requires no attention, as the health does not suffer.

"If only one bag is discovered, and that is of considerable size, it sometimes, if punctured, does not again fill, as will be seen in several of the cases. But when the enlargement is excessive, when a multitude of bags are produced, when the weight of the swelling becomes several pounds; when the breast is very pendulous, and drags upon the surrounding parts, and shakes upon every motion; when there is great apprehension, on the part of the patient, of some malignant disease, then the surgeon will be wise in removing it.

"The operation itself is a simple piece of dissection, in which it is the best plan to secure each divided vessel in immediate succession, to prevent any great loss of blood; but it must be confessed that this is not absolutely necessary, as the operation does not require much time in its performance, and the vessels can be compressed by an assistant whilst the surgeon is removing the tumor; or, if he prefer it, each vessel may be secured in a ligature as the operation proceeds.

"When the tumor requires removal for this disease, it is necessary to take away all the hardened and swollen parts of the breast, for they have cysts or cells formed in them; and, if any cyst be suffered to remain, it will still continue to grow, and the remaining part of the breast to form an hydatid tumor.

"The great solace to the patient in this disease is, that, as it does not contaminate other structures, there is no danger of its extending by absorption, of its producing any complaint beyond the breast, or of its affecting other parts of the body; nor have I seen it seated in both breasts at the same time." (P. 25.)

Sir Astley relates twelve very instructive cases of these hydatid tumors in the breast. In one, cellulous hydatids were united with a schirrous tubercle, and the lady fell a victim to the disease.—In the first case, the patient was taken into the operating theatre for the purpose of having the tumor removed, but, upon examining it with great attention, Sir A. felt a fluctuation. A lancet was introduced to ascertain the nature of the contents. Serum only was discharged. A small piece of lint was introduced into the orifice; adhesive inflammation was brought on; the sides of the cyst adhered, and the patient did well, having no return of the complaint.—In the second case there was distinct fluctuation, surrounded by a wall of hardness. A quantity of serum was discharged by an opening made with a lancet; adhesive plaster was applied, and the wound healed without further application.

The second species of hydatid disease in the breast is of a very curious nature. Its peculiar appearances can scarcely be conveyed by verbal description. Sir A. gives a plate of it, taken from a tumor in the breast of Mrs. King.

"The breast was in this case enlarged, and in the greater part hardened by the effusion of fibrine (coagulable lymph) in lobes into the cellular tissue; but in several parts it contained bags of serum, and formed fluctuating cysts of various sizes. In each of these cells there hung a cluster of swellings, like polypi, supported by a small stalk; and the little pendulous projections appeared to float in the fluid which had been produced around them in the different cysts. Many hydatids were found in a detached state, both in the fluid within the bags and in the solid effusion in the breast; and, taking the whole tumor, vast numbers of them had been formed in it. Their size varied, but the largest did not much exceed that of a barleycorn, the figure of which they assumed. In general they were of an oval form, or I ought to say oviform, as they were larger at one end than the other.

"When opened, they were found to be composed of numerous lamellæ, like the crystalline humor of the eye, or like the layers in the onion, which could be readily peeled from each other. When removed from the breast they had a pearly appearance, and the laminated character of pearl internally.

"The cyst in which they were contained was a perfect bag, and it was composed internally of a membrane which was highly vascular, like other secreting surfaces; and the solid part surrounding the cyst had a greater number of vessels near the bag than at a remote distance from it; but the whole of the diseased structure was endowed with great vascularity." (P. 41.)

In its external character this species resembles the first which has been described. The absence of tenderness being the same in both, it will thus be distinguished from the simple chronic disease of the breast. It cannot be discriminated from the former hydatid disease but by dissection.

"From the scirrhous tubercle it is known by the hardness, by the occasional severe pain, and by the broken health which usually attends that disease; for, although in the case from which I have given the description of this complaint the tumor weighed thirteen pounds upon its removal, yet the general health was good, the absorbent glands in the axilla were unaffected, and there was no local disease in any part of the body. It may be also observed, that scirrhous tumors very rarely acquire so great a magnitude as the hydatid swelling here produced." (P. 43.)

Extirpation is the only mode of relief; neither constitutional remedies nor local applications are of any service.

The third species of hydatid which is found in the breast is the animal or globular. It consists of a bag containing a fluid which has no vascular connexion with the surrounding parts, and it produces within its interior a multitude of bags similar to itself. They are often met with in great numbers in the liver, and have been frequently seen in the lower part of the abdomen, between the bladder and rectum, where they have been the cause of retention of urine. The lungs are sometimes the seat of this species of hydatid. Sir Astley is induced to believe these hydatids "to be distinct animals:

"First, because they have an existence and growth of their own, having no vascular connexion with the part in which they are found, but being only encased and surrounded by a vascular and secreting cyst. Secondly, because they have the power of producing upon their interior surface their own species. Thirdly, that in the brain of sheep a similar bag is found, which, for several hours after the sheep has been killed, if it be put into warm water, has a distinct and very considerable vermicular motion; and, fourthly, because, on the surface of the abdominal viscera, and sometimes in their interior, an hydatid is found with a mouth and neck added to it, and consequently receives its food through the mouth, like other animals.

"The globular hydatid, therefore, may be considered, as to its mode of nourishment, the link in the creation between the animal and vegetable, as it receives its nutriment by absorption as the vegetable does; but the tænia hydetigina, as it is called, which has a mouth, is a perfect animal, with respect to the manner of its nutrition.

"The hydatid is supposed to be deposited in the structure in which it grows, carried there by the blood. Into whatever part it is thrown, it excites irritation, and becomes enclosed by an adhesive process, and which forms the cyst in which it is enveloped; but their origin is obscure, and the opinions respecting their deposition hypothetical. The parent hydatid is supported by a secretion from the internal surface of the cyst in which it is found; but the small hydatids in it are probably nourished by the fluid which the parent hydatid contains, so soon as they drop from, and cease to be connected with the parent cyst." (P. 47.)

The proper treatment of these hydatid tumors is to make an incision in them, and to discharge the bag, after which a simple poultice will be sufficient to heal the wound. If the fluid is discharged by puncture, and afterwards reaccumulates, a seton may be passed into it, and the sac will slough. "When the fluctuation escapes observation, and the tumor is believed to be of a scirrhous nature, the surgeon removes it, and discovers the hydatid bag contained within; and he can then confidently assure the patient that she is perfectly free from any future danger."

The distinguishing marks of this disease are its central fluctuation, its solid circumference, and the absence of tenderness upon pressure. Neither before nor after operation is it dangerous.

Chapter iv. "*On the chronic Mammary Tumor.*"—This disease mostly attacks young persons, from the age of seventeen to thirty. They are generally otherwise healthy subjects, and the constitution does not usually suffer from this local malady. The symptoms which accompany this swelling are, that it grows from the surface of the breast, rather than from its interior. It therefore generally appears to be very superficial, excepting if it spring from the posterior surface of the breast, when it is deep seated, and its peculiar features are less easily discriminated. Such tumors are very moveable, generally not tender to the touch, but Sir A. observes that they are so occasionally at about the period of menstruation. Their growth is slow. They rarely acquire a considerable magnitude, usually weighing from one to four ounces. They are free from malignancy. They sometimes exist for many years almost in a stationary state, and then gradually disappear.

"Upon a nice manipular examination of this swelling, it is found to be lobulated, that is, composed of a number of lobes connected together, but leaving depressions between them; and, whatever size it may obtain, it still preserves this conglomerate character: the swelling might therefore very properly receive the name of the lobulated mammary tumor." (P. 53.)

*Diagnosis.*—The general discriminating marks of this disease are as follows: the youth of the patient, the general absence of pain, the continuance of good health, the slow progress of the tumor, its superficial situation, its extreme mobility, and, above all, "it is known from its lobulated feel, being distinctly composed of numerous lobes conglomerated into one mass, with a broken or divided surface."

In such cases the general health, if disturbed, is to be carefully regulated. If the digestive functions are imperfectly performed, mercurial alteratives, and light bitters with alkalies, will be beneficial. Locally, the Emplast.

Amm. cum Hydr. may be applied, if the part is completely indolent; or iodine ointment may be applied by friction upon the swelling. If the tumor is hot or painful, evaporating lotions or simple poultices are most productive of relief. Several cases of this form of disease are detailed.

In conclusion Sir Astley observes, that, "although these tumors are not in their commencement malignant, and they continue for many years free from the disposition to become so, yet, if they remain until the period of the cessation of menstruation, they sometimes assume a new and malignant action."

In Chapters v. and vi. we have brief descriptions of the "*Cartilaginous and Ossific Tumor*," and the "*Adipose Tumor*."

Chapter vii. "*On the large and pendulous Breast.*"—The glandular structure of the breast sometimes grows to an enormous size, and becomes extremely pendulous, so as to reach to the fore part of the abdomen. This is not to be understood as the effect of relaxation, but to be an absolute growth of the secreting lobes, which can be distinctly felt to be enlarged and hardened, and are sometimes accompanied by a considerable degree of tenderness. Sir Astley has seen a very remarkable instance of this kind. The patient was fifteen years of age, and in good health: for three years the breasts had been increasing in size; she had menstruated irregularly. When Sir A. was applied to, the mammæ were of the following extraordinary dimensions: the circumference of the left, twenty-three and a half inches; of the right, twenty-two inches. They were pendent like a pear. Mr. Jones, of Haverfordwest, who transmitted a description of the case to the author, could perceive no tumor either in the breast or in the axilla. The patient was free from pain.

"The local treatment of this case consists in the application of a suspensory bandage from the back of the neck, under each breast, to produce artificial support; and the principle which is to be observed in the constitutional treatment of this malady, is to increase and to support the menstrual secretion; and for this purpose the exhibition of different forms of steel, united with aloes, will be found the most efficacious medicine. The Ferrum Ammoniatum, the Mistura Ferri composita, the Carbonate of Iron, will be the forms of steel which, united with aloes, will be most beneficial; and, if the biliary secretions be defective, the Pil. Hyd. Sub. Comp., or the Hyd. cum Creta, will be the best medicines." (P. 71.)

Chapter viii. "*On the Scrofulous Swelling of the Breast.*"

Sir Astley has sometimes, though rarely, seen tumors of a scrofulous nature form in the bosoms of young women who had enlargement of the cervical absorbent glands. These swellings are in most cases confined to a single tumor in one breast; but, in one case, two existed in one breast, and one in the other. They are entirely unattended with pain, are distinctly circumscribed, are very smooth on their surfaces, and scarcely tender to pressure. They are very indolent, and vary with the state of the constitution, diminishing as it improves, and increasing as the general health is deteriorating. They can only be distinguished from the simple chronic inflammation of the breast by the absence of tenderness, and by the existence of other diseases of a similar kind in the absorbent glands of other parts of the body. They produce no dangerous effects, and do not degenerate into malignancy. They require no operation.

"The treatment in this case consists in improving the constitution by a warm and dry atmosphere, by an equally regulated temperature, by tepid sea-bathing, by gentle and regular exercise, by animal food of the most digestible kind, by milk, and by a farinaceous diet; a diet which shall nourish without exciting feverish heat, or calling much upon the powers of digestion. The best medicines are Carbonate of Iron and Rhubarb; the Hydr. cum Creta with Rhubarb; a grain of blue Pill, and two or three grains of Quinine; Infusion of Calumbæ with Rhubarb and Soda: for I conclude it will be admitted by every one who deserves the title of a surgeon, that we possess no specific remedy for this disease, but that we are required to assist the digestive powers, make better blood, and convey it to the system by an increased vigor of the constitution.

"Local treatment avails but little: a stimulating plaster or a lotion to the tumor, when the health is improved, may excite the absorbents to remove it." (P. 74.)

Chapter ix. "*Of the Irritable Tumor of the Breast.*"—The breast is liable to become irritable without any distinct or perceptible swelling, as well as to form an irritable tumor, composed of a structure unlike that of the gland itself, and which therefore appears to be of a specific growth.

"When the complaint affects the glandular structure of the breast, there is scarcely any perceptible swelling, but one or more of its lobes becomes exquisitely tender to the touch; and, if it be handled, the pain sometimes continues for several hours. The uneasy sensation is not confined to the breast alone, but it extends to the shoulder and axilla, to the inner side of the elbow, and to the fingers; it also affects that side of the body even to the hip. The patients cannot sleep on that side, and the pain is sometimes so severe as to prevent even their resting on the diseased side; and the weight of the breast in bed in some instances occasions intolerable pain." (P. 76.)

When the pain is most severe, the stomach suffers, and vomiting is produced. The suffering is much increased prior to menstruation. There is no external mark of inflammation. This painful state remains for months, and even for years, with little intermission; but it has no malignant tendency. As there is no distinct tumor, an operation cannot be thought of.

"Besides this irritable and painful state of a whole or part of the breast, a tumor sometimes is found distinctly circumscribed, highly sensitive to the touch, acutely painful at intervals, more especially prior to menstruation, very moveable, often not larger than a pea, seldom exceeding the size of a marble: generally one only exists, but in other cases there are several similar swellings. Although they continue for years, they vary but little in size. I have never seen them suppurate: they sometimes spontaneously cease to be painful, and sometimes disappear without any obvious cause." (P. 77.)

The diagnosis of this disease is very easy. The pain by which it is accompanied, its tenderness to the touch, the suffering which succeeds examination, distinguish it from the hydatid, the chronic mammary tumor, and the scirrhous and fungous tubercle. In such cases Sir Astley has seldom known the menstrual secretion regular or healthy.

The treatment consists in diminishing general irritability, in lulling the local suffering, and in restoring the defective or diminished menstruation. These irritable tumors sometimes appear in other parts, and produce symptoms similar to those which arise when they are seated in the breast. The following case is related as an example:

"Miss B—, a patient of Mr. Brock, of Guernsey, had twice felt a severe pain in her knee in walking, at a considerable interval. Six weeks after the last attack, she discovered a little tumor, about the size of a pea, below the knee, which was extremely painful on the slightest touch: this I removed nine years ago. Twelve months afterwards she discovered, a few inches lower down in the limb, another swelling, which gave the same impression to the finger as the former, but it was more visible, as it projected the skin more; and it produced (as she expressed it) a scraping and pricking pain, as if numerous lancets were darted into the part, and as if all kinds of pain were there combined. It fortunately lasted only ten minutes at each attack; for, if it had continued longer, it would have been intolerable.

"The second tumor I removed eight years ago; and I had the pleasure of seeing her in October last, at which time she had not had any return of the disease." (P. 84 )*

Chapter x. "*On Ecchymosis of the Breast.*"—Allied to the irritability of the breast is a morbid change, which occasionally happens, of a bruised appearance upon this organ. It occurs at each menstruation, and is accompanied by exquisite sensibility, pain, and tenderness.

"The symptoms of this complaint are as follow: It occurs in girls who are in most instances under twenty-two years of age. It is preceded by severe pain in the breast and arm. The extravasation of blood begins a few days before menstruation, and it appears principally in a large spot, as if a severe blow had been inflicted. Smaller and less vivid spots may also be observed in other parts of the breast: it is sometimes a concomitant of an unusually large bosom. The part is exquisitely tender to the touch, and the pain with which it is accompanied passes down along the inner side of the arm to the ends of the fingers. It disappears a week after menstruation in some cases; but in others, when it is most severe, it continues until the next time the patient is unwell. It looks like the ecchymosis which often succeeds the application of leeches; or like the extravasation of blood under the skin which occurs in the arm after bleeding, when the opening in the skin has been smaller than that in the vein.

"It is a curious occurrence, strikingly showing the strong sympathy which subsists between the uterus and breast; for it is evidently the effect of the great determination of blood to the bosom just prior to the period of menstruation; and it indicates excessive irritability of the constitution, as well as the great delicacy and debility of the blood-vessels, which are unable to support this sudden determination which such sympathy produces.

"This complaint is entirely unattended with danger; but being accompanied with diminished, irregular, and sometimes profuse uterine secretion, and by considerable debility and irritability of the constitution, two objects must be kept in view in its treatment: the one is, by different forms of steel medicines, to increase the quantity, and render regular the menstrual discharge; and the other to augment the strength of the system, by the infusion of roses with sulphate of quinine. As to local treatment, the best application is the Liquor Ammoniæ Acetatis, with spirits of wine, in the proportion of five ounces of the former and one of the latter." (P. 85.)

The remainder of the volume is occupied by the splendid plates, and the accompanying descriptions. Although we

* A very similar case is mentioned by Mr. Spark, of Newcastle, in the Medical Gazette, No. 65. The sufferings of the patient were excessive. The tumor was extirpated, and no pain was afterwards felt. The wound healed by the first intention.—Rev.

have been desirous of giving a very full abstract of the text, it must be evident that the work derives a great part of its utility from the instruction imparted by the graphic illustrations. For the purpose of securing a faithful representation of the morbid parts, Sir Astley has always placed them in the hands of the artist as soon as possible after their removal from the patient. In every respect the plates are beautifully and skilfully executed, and, if the colouring be compared with the appearances of recently dissected parts, it will be found correct and natural.

We trust that this work will soon be completed by the publication of the second part. There are no diseases in the management of which surgeons of ordinary experience feel greater perplexity or responsibility than those which affect the female breast. The great advantage to the whole profession of possessing the opinions of Sir Astley Cooper upon so important a subject must be very evident.

---

*A Treatise on the Diseases of the Chest, and on Mediate Auscultation.* By R. T. H. LAENNEC, M.D. Regius Professor of Medicine in the College of France, Clinical Professor to the Faculty of Medicine of Paris, Physician to her Royal Highness the Duchess of Berri, &c. *Translated from the latest French Edition, with Notes and a Sketch of the Author's Life,* by JOHN FORBES, M.D. Member of the Royal College of Physicians, and senior Physician to the Chichester Infirmary. With Plates. Third Edition revised, with additional Notes.—8vo. pp. 736. Underwood, London, 1829.

THE rapid sale of this work is a proof of the high esteem with which LAENNEC is regarded in this country. Dr. FORBES, also, claims much more than the merit of a laborious and verbal translator. He has made many very judicious alterations and improvements upon his original, and has enriched the present edition with many additional notes. The whole work, indeed, has been carefully revised. But little more than a year has elapsed since the publication of the second edition: we then devoted three articles to the consideration of the first part of the work,* and shall now confine ourselves to the second part, which is equally important, although it is much more brief.

In this division the subjects considered are "Diseases of the Heart and its Appendages." So lately as the close of the last century, affections of the heart might still be classed among those diseases which were most imperfectly known.

* London Med. and Phys. Journal, for February, April, and May, 1828.

Notwithstanding the labours of LANCISI, MORGAGNI, &c. ordinary practitioners knew of no other cases than those of polypus of the heart, (an imaginary disease in their acceptation of the term,) and palpitation, (which they considered to be a nervous affection.) The researches of the above-mentioned pathologists, and those of CORVISART, made us acquainted with many organic lesions of the heart, but threw little light on their signs. It was still, perhaps, impossible to distinguish with certainty one disease from the other.

The positive signs of the organic diseases of the heart are derived partly from percussion, but chiefly from auscultation. The common, yet vague, symptoms arising from functional disturbance acquire also by the same means a much greater degree of certainty. The application of the hand, the only method in use before the time of AVENBRUGGER, furnishes us, in most cases, with no result whatever, and frequently deceives us in respect of the actual force of the heart's impulse or shock. It indicates less accurately than the pulse at the wrist, the regularity or irregularity of its contractions.

"Even percussion supplies us with only accessory or corroborative signs, which may frequently be wanting. In reference to this mode of exploration, we must notice two precordial regions, the right and left: the first comprising the space covered by the lower third of the sternum; the second, that which corresponds to the cartilages of the fourth, fifth, sixth, and seventh sternal ribs. The right precordial region naturally yields a very clear sound. Hypertrophy of the ventricles, the dilatation of these or of the auricles, a vast accumulation of blood in all the cavities of the heart, the growth of much fat around this organ, and effusions into the pericardium, may render the sound dull or dead. The same causes may produce the same effect in the left precordial region; but in this case the sign would be less conclusive, inasmuch as this region naturally yields but little sound in most persons, and hardly any in fat or œdematous subjects, or even in such as are very muscular. It is very uncommon for the sound to be wanting in either region, as high as the site of the auricles; and, if it is so, it indicates an enormous dilatation, such as exists only in the case of contraction of the mitral orifice." (P. 546.)

The alternate contractions of the auricles and ventricles of the heart give rise to sounds very distinct, and of different kinds, so as to enable us to study the actions of that organ even more exactly than by the dissection of living bodies. The truth of this apparently paradoxical assertion rests on the fact that the ear judges more correctly of the intervals of sound, than the eye of the intervals of motions corresponding to these.

The first book is introductory: it treats of the exploration of the heart by means of the ear alone, or assisted by the stethoscope.

The second book contains a description of diseases of the heart in general. In the first section we have a detail of the symptoms common to all diseases of the heart. The severest and most common are—dilatation of the ventricles, thickening of their walls, or the union of both affections. Most frequently a single ventricle is affected; sometimes both are so in a similar or in an opposite manner, as in the common case of dilatation of the right ventricle with hypertrophy of the left, and vice versa. The persistence of the foramen ovale, the perforation of the septum between the ventricles, the ossification of the sigmoid valves of the aorta or of the mitral, excrescences on the same parts, and accidental productions formed in the heart, are of much rarer occurrence, and do not, generally speaking, impair the health, until they have reached such a degree as to give rise to hypertrophy, or dilatation of the ventricles. The dilatation and hypertrophy of the auricles are rarer still, and are perhaps always consecutive affections, depending on previous disease of the valves or ventricles. The general symptoms of all these affections are almost the same.

"These are, an habitually short and difficult respiration; palpitations and oppression, constantly produced by the action of ascending, by quick walking, by emotions of mind, or without any perceptible cause; frightful dreams, and sleep frequently disturbed by sudden starts; a cachectic paleness, and a tendency to anasarca, which, indeed, comes on after the disease has persisted some time. To these symptoms is frequently added the angina pectoris, a nervous affection, which will be described hereafter. When the disease has reached a high degree, it is recognised at a single glance. The patient, unable to bear the horizontal posture, remains night and day sitting rather than lying in his bed, with the face more or less swollen, sometimes very pale, but more commonly of a deep violet tint, either over the whole or only on the cheeks. The lips are swollen and prominent like a negro's, of a deeper purple than the rest of the face, or of this hue when it is quite pale. The lower extremities are œdematous; and the scrotum or labia, the trunk of the body, the arms, and even the face, are successively affected in the same manner. The same state exists in the serous membranes, whence arise ascites, hydrothorax, and hydropericardium, which accompany organic affections of the heart more frequently than any other disease. The congestion and lentor of the capillary circulation are further shown by affections of the internal organs: for instance, hæmoptysis, pains of the stomach, vomiting, apoplexy (which frequently terminates such affections), and, most of all, dyspnœa, which last symptom has been the cause of confounding such diseases, with many others, under the name of asthma. These symptoms, however, as they show themselves in the diseases of the heart, have peculiar characters, which tend to distinguish them from such as occur in the affections most likely to be confounded with them.

"In the diseases of the heart the general circulation is not always so much affected as the capillary. Sometimes the pulse is irregular, but sometimes it is almost natural; and the hand, applied to the cardiac region, discovers only a regular and moderate pulsation. At other times the pulse is very strong, or altogether insensible; the heart yields a very great impulse, or none at all, its contractions are evidently irregular, and palpitation is constantly present. So severe a state of disease as this is not always beyond relief: we sometimes see the judicious combination of blood-letting, diuretics, and tonics, remove the impending suffocation, the palpitations, and dropsy, and restore to the patient, frequently for a long period, a tolerable degree of health; and it is commonly only after a great many similar attacks, recurring after considerable intervals, that the disease at length proves fatal." (P. 589.)

Dr. Forbes correctly observes, that this epitome of the general symptoms, as given by Laennec, is excellent as far as it goes, but it must be admitted that the paramount importance of the auscultatory diagnostics on his mind has rendered his description too brief. Dr. F., therefore, directs the attention of the reader to the ampler details on this subject in the classical works of Corvisart, Testa, and Kreysig. He remarks, also, that Laennec has hardly noticed one class of symptoms which merit particular consideration, those referrible to disordered or diseased stomach. The three distinguished writers just mentioned, and especially Testa, notice this state of the stomach at some length; "but none of these authors consider it in its highly important etiological relations, and its still more important bearings on the treatment of the cases in which it occurs. Gastric irritation, cerebral irritation, cardiac irritation, constitute, in many cases, such a strong chain of disease, every part of which influences and strengthens every other part, that no plan of treatment that does not embrace the whole can be attended with success." (*Translator.*)

Dr. Forbes has frequently verified the truth of a remark of Kreysig's, (sect. iii. chap. vii.) that the assumption of a posture in bed, which was previously intolerable, is a sign of extremely bad omen. He has met with several cases of convulsions apparently depending on disease of the heart.

Sect. ii. "*Of changes produced by diseases of the heart in the texture of other organs.*"—In persons who have died from organic diseases of the heart, we find, upon dissection, all the marks of congestion of blood in the internal capillaries. The mucous membranes, especially those of the stomach and intestines, are of a red or violet tint; and the liver, lungs, and capillaries situated beneath the serous, mucous, and cutaneous tissues, are gorged with blood. The augmented colour of the mucous membranes varies much in degree and extent. Sometimes it is observed only here and there, under the form of small points or specks, disseminated over the surface of the membrane. At other times it occupies the whole extent of the surface, and has the appearance of being attended with some swelling of the part. These two appearances are sometimes so considerable, that, if we looked to them merely, without examining the condition of the heart, and without reference to the history of the patient, who had been found capable of taking wine and other stimuli, without experiencing pain, even up to the period of his death, we might be tempted to believe that the fatal disease had been a violent inflammation of the stomach and bowels.

"In fact, the degree of redness of these membranes, observed after diseases of the heart, is often much more intense and extensive than is found after true inflammation of these parts, as, for example, in dysentery; a fact, among many others, sufficiently proving the insufficiency of mere redness to characterize inflammation of the mucous membrane of the intestines, any more than the purple colour of the face in asthmatic patients is an erysipelas. In persons who have died of disease of the heart, particularly dilatation of the ventricles, we find, more frequently than in other cases, that intense redness of the inner membrane of the heart and large vessels, which I shall hereafter notice when treating of the diseases of the aorta. Lancisi and Senac, after Hildanus, consider gangrene of the limbs as a consequence of disease of the heart and large vessels. The late M. Giraud was of the same opinion; and since his time many practitioners have considered the gangrene of old persons as usually caused by ossification of the arteries. M. Corvisart justly doubts whether, in such cases, there is any thing else but mere coincidence of independent diseases;* and I think that the single circumstance of the rarity of the spontaneous gangrene of the limbs, compared with the frequency of disease of the heart and ossification of the

* "Testa (tome iii. p. 333,) and Kreysig (sect. iii. chap. vii.) are of the same opinion.—*Transl.*"

arteries, is sufficient to render the thing quite improbable. This is equally the case with the notion of Testa that ophthalmia, and sometimes the loss of the eye, may be ranged among the consequences of diseases of the heart."* (P. 592.)

It is confessed that none of these symptoms or results suffice to indicate, with certainty, disease of the heart: they are common to many other affections, and particularly to almost every chronic disease of the lungs. Neither the pulse nor the action of the heart supply us with any information upon which we can depend. "To mediate auscultation, therefore, we must turn, as affording the only means of recognising the diseases of this organ; and even *it* more frequently fails in this case than in any of the other diseases which it is calculated to discover." It must be remembered that, if we are ignorant of the previous state of the patient's health, which is almost always the case in hospital practice, we may mistake mere nervous palpitations for hypertrophy or dilatation of the heart.

The causes of diseases of the heart are as various as the diseases themselves. Ossifications are the result of some aberration of the process of assimilation, which is not easily understood. All diseases which give rise to severe and long-continued dyspnœa almost necessarily produce hypertrophy or dilatation of the heart, through the constant efforts the organ is called on to perform, in order to propel the blood into the lungs against the resistance opposed to it by the cause of the dyspnœa. Diseases of the heart, on the other hand, give rise to several diseases of the lungs: they are thus amongst the most frequent causes of œdema of the lungs, hæmoptysis, and pulmonary apoplexy. A neglected *cold* is frequently the original cause of the most severe diseases of the heart.

"To all these causes must be added the congenital disproportion between the size of the heart and the diameter of the aorta. M. Corvisart has, perhaps, gone too far in asserting that there can be no dilatation of the heart without the previous existence of a disproportion of this kind, or of a contraction, or some similar obstruction to the circulation, at a greater or less distance from the heart: it is, however, true that it is very common to find an aorta of small diameter in cases of hypertrophy or dilatation. Still this is not always the case, and, however rational such a cause may be, we can readily conceive many others. We know that the energetic and reiterated action of all muscles materially increases their size, as in the case of those of the right arm of the fencer, the shoulder of the porter, and the hands of most artizans. On

* "Op. cit. t. ii. p. 132."

the same principle we must admit that even nervous palpitations, or such as originate from moral causes, may, by frequent recurrence, produce a true enlargement of the heart.

"There is yet another congenital cause of disease of the heart, which appears to me to be of greater frequency than the small caliber of the aorta, above mentioned: I allude to a disproportionate thickness of one or both sides of that organ. I am satisfied that in a great many persons the parietes of one or both sides of the heart are either too thick or too thin from birth. In such cases there can be no doubt that the usual exciting causes, moral and physical, will be more apt to produce formal disease of the heart than in individuals in whom this disproportion does not exist." (P. 594.)

Laennec's account of the causes of the diseases of the heart is certainly imperfect, but Dr. Forbes supplies this deficiency in a long and excellent note.

By hypertrophy of the heart, Laennec means a simple increase of its muscular substance, without a proportionate dilatation of its cavities. On the contrary, these are most commonly much diminished in size. This affection is by no means common. It appears to have escaped the notice of Corvisart; for he seems to consider that increased thickness of the walls is uniformly accompanied by a proportionate dilatation of the cavities of the organ. Bertin, in his "Traité des Maladies du Cœur et des gros Vaisseaux," has taken much pains to prove the separate existence of hypertrophy and dilatation of the heart. Laennec did not conceive that he was the first to point out the distinction in question, although he was not aware that Bertin had made such extensive researches upon the subject. Hypertrophy may exist in one or both ventricles, with or without a similar affection of the auricles. The auricles alone are sometimes affected in this manner.

"When affecting the left ventricle, I have seen its walls more than an inch, or even eighteen lines, thick at the base, that is, double or triple their size in the sound state. Commonly, this morbid thickening diminishes insensibly from the base to the apex of the ventricle, where it is scarcely perceptible: sometimes, however, the apex partakes in the enlargement, as I have seen it from two to four lines thick, which is double or quadruple the natural size. The columnæ carnæ of the ventricles and the pillars of the valves acquire a proportionate enlargement. The septum between the two ventricles becomes also considerably thickened in the disease of the left ventricle, (which fact seems to mark it as belonging to this rather than the other ventricle,) but in general not so much so as the other parts. There are, however, exceptions, as we find, (and this has been well remarked by M. Bertin,) that the hypertrophy is sometimes unequal in each part of the ventricles, or occupies only a single point, as the base, apex, or middle, the septum or loose part, the external surface or fleshy columns. The muscular substance in these cases is of a degree of consistence sometimes double the natural, and is of a redder colour. The cavity of the ventricle appears frequently to have lost in capacity what its walls have gained in thickness. Sometimes I have found this so small, in hearts twice the size of the fist of the individual, as scarcely to be capable of containing an almond in its shell. The right ventricle in such cases being proportionably smaller as the hypertrophy of the other is great, lies flattened along the septum, and does not extend to the apex of the heart. In extreme cases, it seems as if it were merely included within the walls of the left ventricle." (P. 598.)

When hypertrophy of the right ventricle occurs, the appearances are somewhat different. The thickening is more uniform, and never so great as in the left. Laennec has never found it greater than four or five lines.

We must refer to the work for the signs of hypertrophy of the ventricles.

To hypertrophy of the left ventricle, Corvisart applied the term of *active aneurism* of the heart. The same pathologist named a dilatation of the cavities of the ventricles, with decreased thickness of their walls, *passive aneurism.* Dilatation with hypertrophy of the ventricles are sometimes combined, constituting the *active aneurism* of Corvisart. This form of disease, Laennec states, is much more common than simple dilatation, and still more so than hypertrophy without dilatation. This complication may exist in one or both ventricles. "In the latter case, the heart acquires a prodigious size, sometimes more than triple that of the hand of the individual. The augmentation of volume is here the effect of thickening of the walls of the ventricles, and proportional enlargement of their cavities."

*Signs.*—The signs of this affection are a compound of those of hypertrophy and dilatation.

"The contractions of the ventricles yield at the same time a strong impulse and a very marked sound. Those of the auricles are also sonorous. The sound of the heart's action is heard over a great extent; and sometimes, particularly in thin subjects and children, even the shock is perceptible below the clavicles, on the sides, and even a little on the left side of the back. In the case of a woman who laboured under this affection, I heard and felt the contraction of the ventricles at the right and lower part of the back; and, although this patient was of a small stature and middling strength, the impulse and sound, in the places mentioned, were greater than in the region of the heart in the case of a strong man in perfect health.*

"In this affection, the contractions of the ventricles are very easily perceived by the hand; which (particularly during palpitation) is moreover forcibly raised by the sharp, definite, and violent pulsations. Even in the absence of palpitation, if we attentively observe the patient, we frequently perceive the head, limbs, and even the bedclothes, strongly shaken at each contraction of the heart. The pulsations of the carotid, radial, and other superficial arteries, are frequently visible. If we press on the region of the heart, this organ, according to the expression of Corvisart, 'seems to be irritated by the pressure, and beats more forcibly still.' To these energetic contractions of the heart, according to this author, corresponds (when the disease affects the left ventricle) a pulse which is frequent, strong, hard, vibrating, and difficultly compressed. This state of pulse is, no doubt, frequently met with in hypertrophy with dilatation, as well as in simple hypertrophy of the left ventricle; I cannot, however, consider it, with Corvisart, as a sign of the active aneurism of the left ventricle, inasmuch as we very frequently observe a small and feeble, although regular, pulse, in subjects whose hearts are much enlarged and habitually violent in their action. The palpitations which take place in this affection present, under the stethoscope, the same characters as the habitual contractions in the same case, only in a more intense degree; they are seldom attended with irregularities, except on the approach of death. Sometimes, during these palpitations, besides the impulse of the heart, which seems communicated by a large surface, we can distinguish another which is sharper, clearer, and shorter, although occurring at the same time, and which seems to strike the walls of the chest with a much smaller surface. This blow seems evidently occasioned by the apex of the heart. The examination of the actions of the heart, first on the one side, and then on the other, (that is, under the lower part of the sternum, and between the cartilages of the fifth and seventh ribs of the left side,) enables us to ascertain precisely which of the ventricles is affected, if there is only one; or if they both are so, which is more commonly the case. Dilatation with hypertrophy, being, of all the affections of the heart, that in which this organ attains the largest size, it is in this, accordingly, in which the absence of the natural sound on percussion of the cardiac region is observed most frequently and most extensively." (P. 608.)

* "A singular case of pulsation in the right hypochondre, in a case of diseased heart, is recorded by Mr. Ward, in the London Med. and Phys. Jour. No. 291; in which the pulsation was owing to the right lobe of the liver, enormously enlarged, extending into the chest, and coming in contact with the heart. Many of the cases of pulsation felt very remote from the heart, may be explained by the intervention of a conducting medium superior to that which naturally exists in these situations; although this result arises also from many other causes. See 'Original Cases,' p. 137, 150.—*Transl.*"

In the next chapters, dilatation of one of the ventricles with hypertrophy of the other; dilatation and hypertrophy of the auricles; and partial dilatation of the heart, are described.

*Induration of the muscular substance of the heart.*—It has been already observed, that, in hypertrophy of the heart, the muscular substance possesses an unusual degree of firmness and consistence. Corvisart has seen this so great, that the heart sounded like a dice-box when struck, and the scalpel experienced great resistance in cutting it, and produced a peculiar creaking sound. The muscular substance of the heart, however, retained its natural colour, and did not appear to be converted either into the bony or cartilaginous tissue. Laennec is of opinion that this species of induration is extremely rare. He never met with a case of it, although Corvisart states that he has seen several. He observes that the ventricles, in a state of hypertrophy, always yield the *box sound* mentioned by Corvisart, in a degree proportioned to the degree of the hypertrophy. The author cannot admit, with Bertin, "that the induration of the heart may be considered as the first stage of the ossification, since there exist none of the anatomical characters of the transition of one of these states into the other. Induration usually occupies the whole of one ventricle, while ossification affects only a small portion of its walls, and, as we shall see hereafter, rarely attacks the muscular substance. If to these reasons, deduced from simple observation, we wish to add any arguments drawn from theory, it may be stated that induration supposes an increase of nutrition, and ossification a perversion of the nutritive action." (P. 619.)

*Softening of the muscular substance of the heart* is recognised by the flaccidity of the organ, which, at first sight, looks as if withered, and it is found to be easily torn. The softening is sometimes carried so far that the muscular fibre is almost friable, the compressing fingers passing easily through the parietes of the ventricles.

"In this case, whatever may have been the patient's disease, the heart appears only half filled with blood and flattened, and the ventricles equally collapse, whatsoever may be their varying thickness. This affection of the heart is almost always attended by some change of colour in the organ. Sometimes this is deeper, and even quite violet; and this is particularly the case in severe continued fevers. More commonly, however, the softening of the heart is attended by a striking loss of colour, so as to resemble the palest dead leaf. This pale or yellowish tint does not always occupy the whole thickness of the heart; sometimes it is strongly marked in the central portions, and very little on the exterior or interior surfaces. Frequently the left ventricle and the interventricular septum exhibit this appearance in a marked degree, while the right ventricle retains its natural colour, and even a degree of firmness greater than natural. Again, we sometimes find here and there spots of the natural colour and consistence in hearts which are, every where else, much softened and quite yellowish. This variety of yellowish softening is particularly observable in hearts of good proportion, and in those cases where dilatation is conjoined with a slight degree of hypertrophy. It is also found in simple dilatation, although it is more common to find this state accompanied by that species of softening which is marked by an augmentation of the natural colour of the organ. There is a third variety of softening of the heart, which will be noticed in another place, and which is attended by a pale white colour of the muscular substance. In this the degree of softening never reaches that of friableness; often it is scarcely perceptible; but the parts are flabby, and the walls of the ventricles quite fall together on being opened. This species of softening usually accompanies pericarditis, and is observed only in it." (P. 6[illegible]0.)

Softening of the heart not having hitherto engaged the attention of practitioners, Laennec remarks that it is very difficult to determine its degree of danger or its distinctive signs. M. Bouillaud considers softening of the heart as a consequence of inflammation, and looks upon the induration, as well as the increase or diminution of colouring of the heart, in the same point of view.

"The only proof brought in support of this opinion is this, that the muscles, the brain, liver, lungs, kidneys, and spleen, become soft when affected with inflammation. In respect of this, I would remark that the reasoning is here in a circle; since it ought to be previously proved that the softening of these organs, when existing alone and without pus, is the consequence of inflammation. On the other hand, if softening of the heart is the consequence of inflammation, this inflammation must be either some degree of that which produces pus, or one of quite a different kind, and having no tendency to produce this. On the first hypothesis, softening of the heart is so common an affection, that we should, sometimes at least, find it arrived at the stage of purulent infiltration: but this state I have never seen, even in the case of softening that has reached so far that the muscular substance yields between the fingers like paste; the muscular fibres still retain their form, and present no trace of pus in their interstices; and I am not aware that pus has been found by any one in such cases. If, on the second supposition, softening of the heart is an affection of such a nature that it tends neither to the formation of pus, nor is attended by local pains, nor any of the local and general symptoms which constitute inflammation; if the therapeutic measures found beneficial in inflammation are directly the reverse of those which the state of the individuals usually affected with softening of the heart seems to demand; why give the same name to affections so different?" (P. 622.)

Softening of the heart, in the opinion of Laennec, "is a disease *sui generis,* produced by some aberration of assimilation, whereby the solid elements of the tissue diminish in proportion as those which are fluid or semifluid increase." With every disposition to respect even the speculative opinions of Laennec, we can discover nothing more in this attempted explanation than that the heart is no longer firm because it has become soft. The author states that we ought to regard it as a general law of the animal economy, that all soft tissues become indurated in consequence of true inflammation; that is, an inflammation tending to the formation of pus. It is only the hard tissues, such as bone, cartilage, and the fibrous bodies, which become softer during inflammation, in consequence of the presence of an increased quantity of plastic lymph of a less consistent quality than that of bone.

The tenth chapter is very brief upon the subject "*of atrophy of the heart.*" The heart, like the muscles of voluntary motion, is clearly susceptible of diminution of size and loss of power, from the influence of all those causes which produce emaciation. This effect is, however, less remarkable in the heart than in other muscles, and does not become perceptible till after a considerable time. The author is not of opinion that diminution of the size of the heart can in any case be considered as a disease. He never observed any symptom which could be attributed to this cause. On the contrary, all persons in whom it was found appeared to him less subject than usual to inflammatory affections and disorder of the circulation.

"*Malformation of the heart*" forms the subject of the twelfth chapter. Dr. Forbes remarks in a note that the best and most complete account of congenital malformation of the heart has been furnished by Dr. FARRE. This subject has excited particular attention in Germany, and has lately formed the subject of several valuable inaugural dissertations: one of the best is that of Dr. HEIN, "De istis Cordis Deformationibus quæ sanguinem Venosum cum Arterioso misceri permittunt."

The heart is sometimes surrounded by an unusual quantity of fat. It does not appear, however, either from the experience of Laennec or Corvisart, that any symptoms can be detected which denote the existence of such an accumulation.

"*Fatty degeneration of the heart*" is occasionally met with.

"This latter is an actual transformation of the muscular substance into a substance possessing all the chemical and physical properties of fat. It is precisely similar to the fatty degeneration of the muscles observed by Haller and Vicq-d'Azyr. I have only met with it in a small portion of the heart at one time, and only towards the apex. In these portions the natural red colour is superseded by a pale yellow, like that of a dead leaf, and is, consequently, nearly the same as that of certain states of softening of the heart. This change of structure appears to proceed from without inwards. Near the internal surface of the ventricles, the muscular texture is still very distinguishable; more externally, it is less so; and still nearer the surface it becomes gradually confounded, both in colour and consistence, with the natural fat of the apex of the heart. In such cases, however, even the portions that still retain most of the muscular character, when compressed between two pieces of paper, still grease these very much. This character distinguishes this species of degeneration from simple softening of the viscus. I have never found rupture of the heart attributable to this change, any more than to the morbid accumulation of fat. It is denoted by no symptoms with which I am acquainted." (P. 638.)

The author has never met with ossification of the muscular substance of the heart. Corvisart found, in the case of a man who died of hypertrophy of the left ventricle, the whole apex of the heart, and more partially the columnæ carneæ of the left ventricle, converted into cartilage. Filling, in an asthmatic subject, found ossification of one of the fleshy columns of the left ventricle.*

Cartilaginous and bony induration of the valves of the heart not unfrequently occurs. The eighteenth chapter contains some interesting remarks on this subject.

"*Of Concretions of Blood, commonly termed Polypi, of the Heart and large Vessels.*"—It was formerly the custom to attribute to the polypous concretions of the heart observed after death, the symptoms which truly depend on the enlargement of that organ. These concretions, however, are very frequently found in persons who have never exhibited any symptoms of disease of the heart: "in truth, they are met with in three fourths of dead bodies." It is even suggested that "perhaps the existing epidemic constitution contributes as much to their production as the particular condition of the individual." Laennec has been led to this notion by

* Hufeland's Journal, b. xv. p. 155.

having found these "polypi," as they are termed, more often, and much larger, at certain times than others. He is opposed to the opinion of Pasta, Morgagni, and others, that such concretions of blood begin to form merely in the last struggles of life. "Many facts prove that these concretions can be formed during life: the phenomena of aneurisms alone prove this; and, besides, we sometimes find veins, and even arteries of considerable size, completely obstructed by concrete fibrine." This argument appears to us not even specious, much less is it satisfactory. The admitted fact that concretions of blood are found in an aneurismal artery will not fairly lead to the inference that the same occurrence will take place in the heart, without some deviation from its natural condition, which could exert a similar influence to that produced by aneurism upon the circulation of the blood. We do not deny that spontaneous coagulation of the blood may take place, "particularly at the very close of life, when the circulation is performed only in an irregular and imperfect manner." We only object to the endeavour to support this still doubtful fact by the phenomena which occur in aneurism.

"When the polypi of the heart are of a large size, I conceive they may be recognised by the stethoscope. In several cases I have prognosticated their existence from the following signs; which, nevertheless, I dare not propound as certain, as they are not founded on a great many facts: In the case of a patient, whose heart had been acting regularly, if the pulsations suddenly become anomalous, obscure, and confused, so as not to be analyzed, we may suspect the formation of a polypus. If the disordered action exists on one side of the heart only, we may consider the thing as almost certain. For instance, if we find the pulsations of the heart under the sternum confused and tumultuous, although the day before they had been regular, we may look upon the formation of a polypus in the right cavities as very probable; and the more so if the contractions of the left ventricle, explored between the cartilages of the fifth and sixth ribs, are more distinct." (P. 655.)

Notwithstanding the contrary opinion of some modern observers, Laennec thinks that inflammation of the inner membrane of the heart and large vessels is very rare. The correctness of his opinion upon this subject he maintains from an examination of the different morbid appearances which have been considered as proofs of the inflammation in question. 1st. Redness of the membrane. The inside of the aorta and pulmonary artery are often found uniformly reddened, as if stained by the blood they contained. This colouring is of two kinds, either bordering on scarlet, or of a brown or violet hue. This colour is quite uniform, as if painted, without any trace of vascularity, only sometimes more intense in one place than another. Sometimes this stain diminishes progressively from the aorta, but frequently it terminates quite abruptly, with irregular edges. Sometimes nearly the whole arterial system presents the same colour. This redness is attended by no sensible thickness of the part, and it entirely disappears after a few hours' maceration. FRANK, who observed it through the whole tract of the arteries, considered it as the cause of a particular and uniformly fatal fever. KREYSIG, BERTIN, and BOUILLAUD adopt the same opinion.

"The first and most natural idea respecting the redness of any part naturally white, is that it is the result of inflammation. But mere redness, without thickening of parts, does not sufficiently characterize this state; while the abrupt termination and exact circumscription presented by the redness in certain cases, seem not easily to accord with the nature of inflammation, and give rather the idea of impregnation by a coloured liquid, which had been poured irregularly over the membrane, or which had only touched it partially, on account of its small amount.

"I am extremely doubtful whether this kind of redness gives rise to general symptoms sufficiently constant or severe to indicate its presence. I have found it in subjects dead of very different affections, and have never been able to foretel its existence by any constant signs. A rather prolonged agony, in subjects still vigorous, yet cachectic from diseased heart or otherwise, has appeared to me frequently to accompany this affection. In cases of this kind the blood is never strongly coagulated, and the body most commonly affords marks of decomposition." (P. 656.)

"I think we must conclude that the redness of the lining membrane of the heart and large vessels cannot, in any case, be considered as proving the existence of inflammation: on the contrary, that we may consider it as being the result of a process taking place in the dead body, or in the last agony, in every case wherein we find it coinciding with a prolonged and suffocative agony, a manifest change in the fluids, and a more or less marked state of decomposition. This is a state of parts to which I wish particularly to call the attention of pathologists, so that they may avoid confounding the causes with the effects of diseases. The discrimination of the congestion of the capillaries from inflammation is often difficult, but it is of the utmost importance that it should be made.* In the case now in question, we may be justified in suspecting inflammation when the redness is accompanied with swelling and thickening of the part, and with an

* For some valuable observations and experiments on the subject of redness of the inner coat of the blood-vessels, I refer to a memoir of MM. Rigot and Trousseau, in the Archives gen. de Med. t. xii. The result of the researches of these gentlemen corroborates the views of M. Laennec.—*Transl.*

extraordinary development of capillaries in the middle coat of the vessel; but I am not sure that even these characters united would prove the existence of inflammation in the case of a body that was considerably œdematous." (P. 659.)

The formation of a layer of coagulable lymph on the inner surface of the heart and vessels is the most unequivocal sign of inflammation of this membrane, and indeed, with the exception of ulceration, is the only certain one.

The remaining chapters treat of *excrescences of the valves and internal walls of the heart*, of *pericarditis*, of *hydro-pericardium*, of *accidental productions in the pericardium*, of *organic affections of the vessels of the heart*, of *the treatment of the organic diseases of the heart*, and of *nervous affections of the heart and vessels* Laennec is opposed to the doctrines of Heberden, Parry, and most physicians of England, Germany, and Italy, respecting the cause of angina pectoris. He states that

"Angina pectoris, in a slight or middling degree, is extremely common, and exists very frequently in persons who have no organic affection of the heart or large vessels. I have known many individuals who have suffered a few very severe but short attacks of it, and had no further return of it. I am even of opinion that the prevalent type of disease influences its development, as I have some years met with it frequently, and hardly at all in others. On the other hand, it is certainly true that this affection frequently coincides with organic diseases of the heart; but nothing proves even then that it depends upon such diseases, inasmuch as they are of various kinds, and as the angina exists without any of them. I have examined several subjects who have laboured under this disease, and in whom there coexisted either hypertrophy or dilatation of the heart; and in none of these did I find the coronary arteries ossified. One of these died suddenly during an attack of angina; and such a result need not surprise us, when so severe a nervous affection coexists (as in this case) with extensive hypertrophy. Dr. Desportes, in a dissertation published some years since, has stated opinions very analogous to mine respecting the nature and seat of this affection: he considers its site to be in the pneumo-gastric nerve." (P. 703.)

Dr. Forbes is entitled to the gratitude of the English reader for the great labour he has bestowed upon this translation of Laennec's invaluable work. Various parts of it we have collated with the original, and can warmly recommend it to the attention of the profession. It is not merely a faithful transcript: the numerous notes of the translator throw additional light upon many subjects of practical importance which Laennec has but cursorily considered. A copious alphabetical index would be an improvement to a future edition.

*Napoleon à Sainte-Hélène. Opinion d'un Médecin sur la Maladie de l'Empereur Napoléon, et sur la Cause de sa Mort. Offerte à son Fils au Jour de sa Majorité.* Par J. Héreau, ancien Chirurgien ordinaire de Madame Mère, et premier Chirurgien de la Impératrice Marie-Louise.—8vo. pp. 228. Paris.

The author's professed motives for writing this work are, a love of truth and an earnest desire to remove the fear of hereditary cancer from the mind of the young Napoleon: but, if these were really his motives, we imagine he would go immediately to the point, and not weary the reader by a tedious and gratuitous refutation of never-believed and now-forgotten rumors of "poisons, stings, and death;" nor disgust him by exaggerated statements of facts, and by copious and paltry abuse of the English government, and of every illustrious character whose energy or prudence conspired to overthrow the enemy of liberty, the great idol of French ambition and vanity. He appears, then, to be instigated by party and selfish views, and being a factious politician, rather than a medical writer, will not long detain our attention.

According to our author, it is still very generally believed in the French provinces, that Napoleon was removed by poison, and that the companions of his captivity were not permitted to return to France until they gave a solemn promise of secrecy. The author does not share this opinion; but he is so weak as to consider it worth refutation, to which purpose he devotes an entire chapter.

In his second chapter, he attempts to prove that Napoleon was not hereditarily liable to cancer; for he contends that the nature of the disease of which his father died is not precisely known; that all Napoleon's brothers and sisters, and their children, are exempt from apparent predisposition to this disease, as also his mother, who is now in her seventy-eighth year, and to whom he bore a great resemblance, intellectual as well as physical, but was totally unlike his father. It would be unreasonable to deny the collective force of these facts; but, in reference to the argument derived from the want of likeness between the father and the son, it may be asked whether we do not often find relations whose mental faculties and physical shape and proportions are dissimilar, while their instinctive propensities, and, it may be presumed, those peculiar arrangements of structure which constitute predisposition to disease, are analogous.

Having settled the question of hereditary cancer in the negative, the author proceeds to inquire "whether the influence of the climate of St. Helena was sufficient to occasion Bonaparte's complaint?" On this subject he displays either great want of candour or unpardonable ignorance, as may be seen in the subsequent passage: "From that moment (a year after his arrival,) the Emperor was quite convinced that this unhealthy island had been chosen by the English government on purpose to destroy him, and that they had calculated that his death would be thus effected quite gradually enough to appear natural."

This imputation is as unfounded as it is illiberal; for St. Helena is so far from being generally considered unhealthy, that people who are taken ill in India not unfrequently repair to that island, in order to recruit their strength. But it was not possible to find any climate on the face of the earth which, without competent exercise, should keep in health a body which had been always in action, and should preserve a mind which had been so long obeyed, and so much excited by great events, free from vexation and remorse at the remembrance of vanished power and foiled ambition, when its daring was no longer available, its range limited, and its pride controlled and humbled. It is, indeed, probable that a reverse of fortune much less sudden, complete, and degrading, would ultimately have undermined the constitution of such a captive, without the deleterious influence of climate, or of any other merely physical cause.

> "But *quiet* to quick bosoms is a hell,
> And *there* hath been thy bane!"

It is with greater reason that M. Héreau inveighs against the restrictions to which Napoleon was subjected; but here, as elsewhere, he exaggerates, distorts, and garbles nearly every statement which he makes.

The medical attendants do not escape without being brought before the all-censuring tribunal of this self-erected judge; and Antommarchi, the Emperor's last surgeon, is not only condemned as destitute of even the lowest degree of medical skill, but likewise reproached as a sacrilegious monster for daring to examine, after the method of Gall and Spurzheim, the cranium of the lifeless hero.

After having commented on the symptoms and treatment of Napoleon's complaint, the author proceeds, in the fourth chapter, to analyze, rather minutely, the descriptions given of the appearances of the body after death: and here he attempts, but in a most unsatisfactory manner, to prove that the disease of which Bonaparte died was chronic gastritis, and not (as was supposed both by his private attendant and by the English surgeons who visited him during his illness, and were present at the inspection of his remains,) a cancerous affection of the stomach. Our author is not equally strong in argument as he is bold in opinion; nor do we think it possible to devise any train of reasoning powerful enough to prove the truth of M. H.'s assertion to any one in possession of the facts contained in both accounts of the inspection of the body after death, the veracity of which cannot be doubted, and which M. H. has neither suppressed nor refuted; but which he must prove incorrect before we can receive his notion as true.

Whoever places the same reliance on the judgment of the six* surgeons whose opinion is expressed in the following passage, as on that of M. H., cannot, we think, agree with the latter in believing that merely chronic gastritis was the disease of which Napoleon died. "Nearly the whole internal surface of the stomach was in a state of cancer, or of schirrus degenerating into cancer." "It was perforated near the pylorus, and contained a quantity of thick dark matter, resembling coffee grounds, and very fetid."

---

### COLLECTANEA.

* The five English surgeons and Antommarchi, by whom the body was examined after death.

